# Supplementary material for: Distribution and predictive performance of the temporal phase of dynamic spot sign appearance in acute intracerebral hemorrhage
Source: PLoS One. 2020 Aug 6;15(8):e0236196. doi: 10.1371/journal.pone.0236196 (PMC7410294; doi:10.1371/journal.pone.0236196)
Supplement: Attachment — Submitted filename: Response to Reviewers.docx [file pone.0236196.s004.docx]

**Manuscript PONE-D-20-11565**

**Response to Reviewers**

Dear Dr. Sherman,

Thank you for giving us the opportunity to submit a revised draft of the manuscript “Distribution and predictive performance of the temporal phase of dynamic spot sign appearance in acute intracerebral hemorrhage” for publication in *PLOS ONE* journal. We appreciate the time and effort that you and the reviewers dedicated to providing feedback on our manuscript. We have incorporated most of the suggestions made by the reviewers. These changes are highlighted within the manuscript. Please see below, in blue, for a point-by-point response to the reviewer’s comments and concerns.

**Reviewer’s Comments to the Authors:**

1. Please ensure that your manuscript meets PLOS ONE's style requirements, including those for file naming. The PLOS ONE style templates can be found at <https://journals.plos.org/plosone/s/file?id=wjVg/PLOSOne_formatting_sample_main_body.pdf> and <https://journals.plos.org/plosone/s/file?id=ba62/PLOSOne_formatting_sample_title_authors_affiliations.pdf>

**Author response**: Thank you, this has been corrected. Changes are trackable in our revised manuscript file.

2. We note that you have a patent relating to material pertinent to this article. Please provide an amended statement of Competing Interests to declare this patent (with details including name and number), along with any other relevant declarations relating to employment, consultancy, patents, products in development or modified products etc. Please confirm that this does not alter your adherence to all PLOS ONE policies on sharing data and materials, as detailed online in our guide for authors http://journals.plos.org/plosone/s/competing-interests by including the following statement: "This does not alter our adherence to  PLOS ONE policies on sharing data and materials.” If there are restrictions on sharing of data and/or materials, please state these. Please note that we cannot proceed with consideration of your article until this information has been declared.

This information should be included in your cover letter; we will change the online submission form on your behalf.

**Author response:** Name and number are included. I confirm this does not alter our adherence to all PLOS ONE policies on sharing data and materials and will add this statement. I have included the disclosure information in our cover letter.

Please know it is PLOS ONE policy for corresponding authors to declare, on behalf of all authors, all potential competing interests for the purposes of transparency. PLOS defines a competing interest as anything that interferes with, or could reasonably be perceived as interfering with, the full and objective presentation, peer review, editorial decision-making, or publication of research or non-research articles submitted to one of the journals. Competing interests can be financial or non-financial, professional, or personal. Competing interests can arise in relationship to an organization or another person. Please follow this link to our website for more details on competing interests: http://journals.plos.org/plosone/s/competing-interests

**Additional Editor Comments (if provided):**

5. Review Comments to the Author

Please use the space provided to explain your answers to the questions above. You may also include additional comments for the author, including concerns about dual publication, research ethics, or publication ethics. (Please upload your review as an attachment if it exceeds 20,000 characters)

Reviewer #1: The authors examined 'spot sign' on dynamic CTA's (arterial vs. venous phase) ability to predict significant hematoma expansion (defined as 6mL or 33% relative volume growth). This is an important topic as clinical predictors will likely be of value when deciding on trial enrollment for potential therapeutics for ICH. The manuscript is well-written, direct, and easy to follow. The intent of the study is clearly stated and the methods for which the authors attempt to answer their hypothesis is rationale and scientifically sound.

The major limitation of the study is the sample size in each group, in particular the venous phase (n=8), which the authors explicitly mention in the discussion. With this low of a sample size it is challenging to draw a firm conclusion, however, the authors appropriately conclude that the study is 'exploratory' and 'predictability of significant HE and clinical outcome MAY differ across the timing of spot sign appearance on dCTA' and 'larger sample sizes are needed to confirm the findings of our study.' The authors collected subjects prospectively but could consider including retrospective cases if dCTA was being used at their center before they started the study to increase the number of subjects.

**Author response:** Thank you for your feedback and suggestion. Because our study was prospective, our REB approval was to only collect the data under the protocol funded by the Heart & Stroke Foundation of Canada. It is unlikely they will allow retrospective data collection outside the original protocol, and would require a full new submission and review. While this is not impossible, it would take considerable time for the review (which is often 6 months at our institution). Furthermore, due to COVID, non-COVID submissions are being triaged as low priority, so we are concerned this would further delay reviews.

A few minor requests:
Median time of follow-up CT is listed in the Table however please comment on your sites/study's customary practice for serial neuroimaging. For example, repeat CT in all cases within xx h, repeat only if clinically worse.

**Author response**: Thank you for pointing this out. This has been included in lines 134-135.

Please describe method used to measure hematoma volume (automated segmentation, abc2, etc)

**Author response:** Thank you, this has been included in lines 135-136.

Reviewer #2: This article demonstrating a trend towards poor outcome in ICH patients with arterial phase spot sign on dCTA when compared to venous cohorts is well received. The small sample size is mentioned, and publication of this article will highlight the need for further study.

**Author response:** We thank the reviewer.
